# Supplementary material for: Natural Killer Cell Function Tests by Flowcytometry-Based Cytotoxicity and IFN-γ Production for the Diagnosis of Adult Hemophagocytic Lymphohistiocytosis
Source: Int J Mol Sci. 2019 Oct 30;20(21):5413. doi: 10.3390/ijms20215413 (PMC6862274; doi:10.3390/ijms20215413)
Supplement: Supplementary file 1 [file ijms-20-05413-s001.pdf]

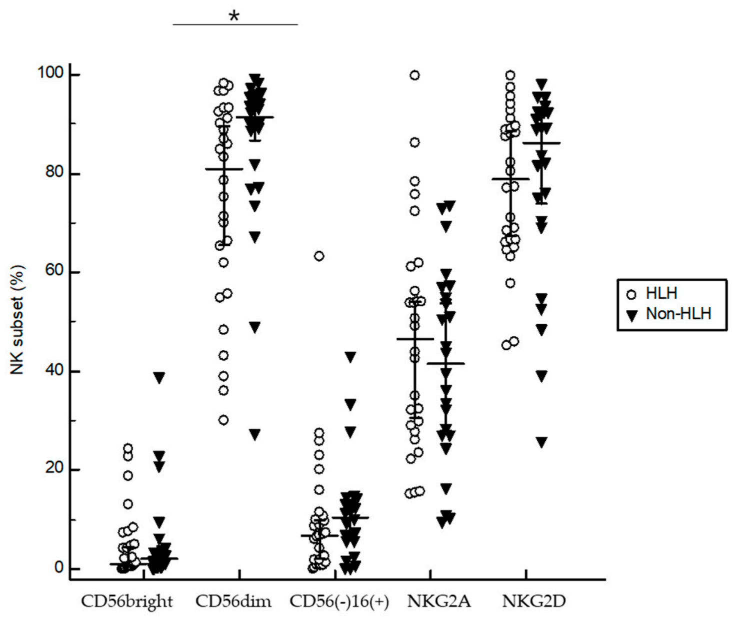

Supplementary Figure 1 Comparison of NK cell subset in patients with HLH and non-HLH patients. CD56dim NK cells (%) decreased in HLH patients compared to those in non-HLH patients [80.9% (65.6-89.7) vs. 91.3% (86.8-94.3), $p = 0.029$]. Percentages of CD56bright, NKG2A (+), or NKG2D (+) NK cells were not significantly different between HLH and non-HLH patients results.

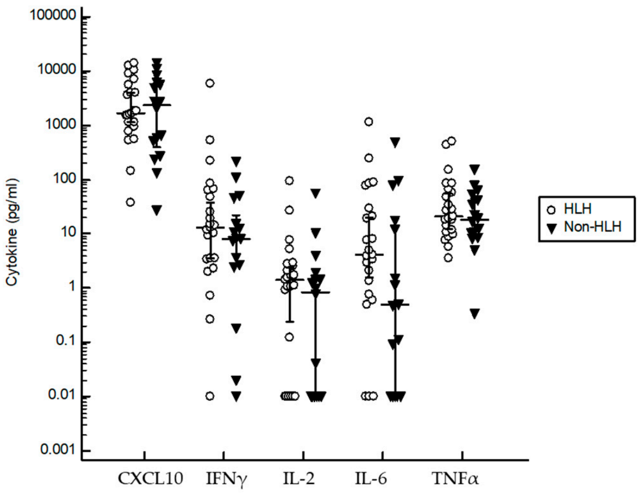

Supplementary Figure 2. Comparison of cytokine results in HLH and non-HLH patients.

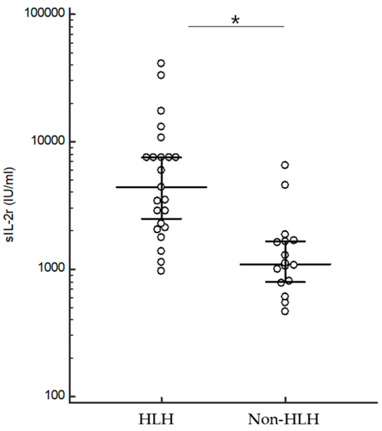

Supplementary Figure 3. Comparison of Serum sIL-2r in HLH and non-HLH patients. Serum sIL-2r level was significantly higher in HLH patients than that in non-HLH patients (4433.0 (2482.7–7500.0) vs. 1098.0 (795.9–1653.2), $p < 0.001$) * $p < 0.05$.

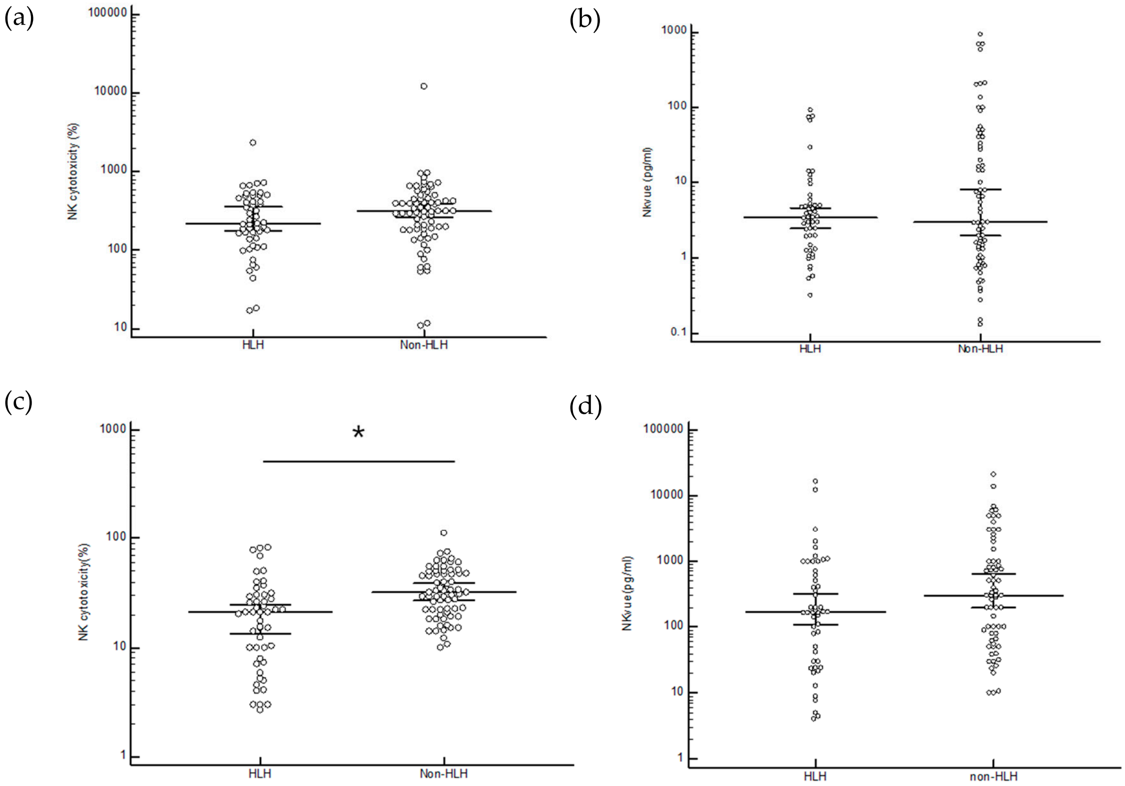

Supplementary Figure 4. Comparison of normalized results of NK-cytotoxicity and NKA-IFNγ based on the NK cells. (**a**) Comparison of NK-cytotoxicity normalized based on NK cell (%) and (**b**) NKA-IFNγ normalized based on NK cell (%) showed no significant difference between HLH and non-HLH patients. (**c**) NK-cytotoxicity showed significant difference in HLH patients compared with non-HLH patients after normalization with CD56dim NK cell (%) (21.0 (13.3–24.6) vs. 31.6 (27.0–38.9), $p < 0.001$). (**d**) NKA-IFNγ showed no significant difference between HLH and non-HLH patient after normalization with CD56bright NK cell (%).* $p < 0.05$.
